# Efficacy and safety of cyclosporine a combined with acitretin in moderate-to-severe plaque psoriasis: a randomized controlled trial

**DOI:** 10.3389/fmed.2025.1667058

**Published:** 2025-09-26

**Authors:** Kang Xu, Ping Yuan, Sujie Jia, Chunyan Gong, Qingjie Hu, Jie Sun, Chao Zhu, Jing Wang, Silu Xu

**Affiliations:** ^1^Department of Pharmacy, Hospital for Skin Diseases, Institute of Dermatology, Chinese Academy of Medical Sciences & Peking Union Medical College, Nanjing, China; ^2^Department of Anesthesiology, Jiangsu Cancer Hospital, Jiangsu Institute of Cancer Research, The Affiliated Cancer Hospital of Nanjing Medical University, Nanjing, China; ^3^Department of Allergy and Rheumatology, Hospital for Skin Diseases, Institute of Dermatology, Chinese Academy of Medical Sciences & Peking Union Medical College, Nanjing, China; ^4^Department of Pharmacy, Tongren Hospital, Shanghai Jiao Tong University School of Medicine, Shanghai, China; ^5^Department of Pharmacy, Jiangsu Cancer Hospital, Jiangsu Institute of Cancer Research, The Affiliated Cancer Hospital of Nanjing Medical University, Nanjing, China

**Keywords:** psoriasis, cyclosporine, acitretin, efficacy, safety

## Abstract

**Background:**

Despite the efficacy of biologics in psoriasis treatment, their contraindications limit accessibility. Traditional systemic agents like cyclosporine A (CsA) and acitretin remain first-line options for long-term management, yet evidence on their combined use is scarce.

**Methods:**

In this randomized controlled trial, patients with moderate-to-severe plaque psoriasis were assigned to CsA + acitretin combination therapy, CsA monotherapy, or acitretin monotherapy. Treatment lasted 12–16 weeks with follow-up to week 24. The primary outcomes were the proportions of patients achieving at least a 75% (PASI75) and 90% (PASI90) reduction from baseline in the Psoriasis Area and Severity Index (PASI). Secondary outcomes included mean change in PASI, Body Surface Area (BSA), Dermatology Life Quality Index (DLQI), and adverse events (AEs).

**Results:**

Of 351 screened patients, 345 were randomized and 305 completed the study. Combination therapy achieved significantly faster and greater responses than monotherapies. At week 4, >60% of patients in the combination group achieved PASI75 versus <25% in either monotherapy arm (*p* < 0.001), and 21.6% achieved PASI90 compared with almost none (*p* < 0.001). These advantages were maintained at week 12 (PASI75, 89.2%; PASI90, 66.7%) and sustained at week 24 (91.2 and 77.5%, respectively). BSA and DLQI improvements paralleled PASI, with greater early benefits in the combination group that converged after week 12. Combination therapy also maintained efficacy with lower mean doses of both CsA and acitretin. Most AEs were mild and reversible: dryness and dyslipidemia were more frequent with acitretin, hypertension with CsA, and hepatic abnormalities higher with combination therapy, though not significant. Overall, combination therapy demonstrated an acceptable safety profile.

**Conclusion:**

CsA–acitretin combination therapy demonstrated superior early efficacy and acceptable tolerability compared with monotherapies while reducing drug exposure. This regimen may represent a cost-effective therapeutic option for patients not eligible for biologic therapy.

**Clinical trial registration:**

https://www.chictr.org.cn, ID register: ChiCTR-OPN-17013383.

## Introduction

Psoriasis is a recurrent, chronic, and inflammatory skin disease that occurs in approximately 3 to 4% of the U.S. population and 0.47% of the Chinese population (1, 2). The disease commonly involves skin, nails, and joints, substantially impairing patients’ quality of life and frequently leading to anxiety, depression, and even suicidal ideation. Consequently, many patients with psoriasis require long-term medical and psychological care (3, 4).

Various treatment modalities are available for psoriasis, including topical therapies (e.g., tacrolimus, calcipotriene, and corticosteroids) (5), phototherapy, non-biological systemic medications (such as methotrexate, acitretin, and cyclosporine) (6, 7), and biological systemic agents (e.g., etanercept, infliximab, adalimumab, secukinumab, and ixekizumab) (8). Biological therapies have become mainstream due to their capacity to induce substantial clinical improvement or even complete remission. However, there are many contraindications to the use of biological agents, such as severe infections, active tuberculosis, hepatitis B, human immunodeficiency virus (HIV) infection, or recent vaccination (9). The choice of treatment for psoriasis is influenced by various factors, including both short-term and long-term responses measured by the Psoriasis Area and Severity Index (PASI), drug efficacy and safety profiles, impact on quality of life, and treatment cost. Traditional systemic agents such as cyclosporine, acitretin, and methotrexate (MTX) remain favorable as first-line therapies due to their affordability, particularly among economically disadvantaged populations globally (10).

To our knowledge, the combination of cyclosporine A and acitretin for severe psoriasis remains controversial (11), and comparative studies evaluating the efficacy and safety of acitretin alone, cyclosporine alone, and their combination therapy are limited. According to psoriasis treatment guidelines, topical therapies or narrowband ultraviolet B (NB-UVB) phototherapy are recommended initially; if these treatments fail to achieve sufficient clinical improvement, systemic therapies such as acitretin, methotrexate, or cyclosporine A, followed ultimately by biologics, should be considered (6). Therefore, this randomized controlled trial aims to assess the efficacy and safety of combined cyclosporine A and acitretin therapy compared to monotherapy with either acitretin or cyclosporine in patients with moderate-to-severe plaque psoriasis. This study intends to provide a cost-effective treatment option for psoriasis patients experiencing financial constraints.

## Methods

### Study design and participants

This randomized controlled trial enrolled patients with moderate-to-severe plaque psoriasis (PASI ≥12) who were diagnosed by dermatologists at the Hospital of Skin Diseases, Chinese Academy of Medical Sciences, between October 2020 and November 2024.

Inclusion criteria were as follows: (1) age 18–80 years, male or female; (2) diagnosis of moderate-to-severe plaque psoriasis, defined as PASI ≥12 or body surface area (BSA) involvement ≥10%; and (3) inadequate response to topical therapies, phototherapy, or both, with no prior use of acitretin, cyclosporine, or biologic agents within the preceding 4 to 6 weeks.

Exclusion criteria were as follows: (1) severe systemic diseases or uncontrolled hypertension; (2) clinically significant hepatic or renal dysfunction; (3) pregnancy, lactation, or planning to become pregnant during the study period; (4) use of systemic glucocorticoids, other conventional immunosuppressants, or biologic agents within 4 weeks prior to enrollment; and (5) known hypersensitivity to acitretin or cyclosporine.

The study protocol was approved by the Ethics Committee of the Hospital of Skin Diseases, Chinese Academy of Medical Sciences, and written informed consent was obtained from all participants prior to enrollment. The trial was registered at the Chinese Clinical Trial Registry (ChiCTR-OPN-17013383).

### Randomization and blinding

Eligible participants were randomly assigned in a 1:1:1 ratio to one of three treatment groups: the combination therapy group (acitretin plus cyclosporine), the acitretin monotherapy group, or the cyclosporine monotherapy group. Randomization was performed centrally using a computer-generated random sequence with variable block sizes concealed from investigators, prepared by an independent statistician who was not involved in patient enrollment or treatment. Allocation concealment was maintained through sealed, opaque, and sequentially numbered envelopes. Because of the distinct dosing schedules and monitoring requirements of acitretin and cyclosporine, participants and treating investigators were necessarily aware of treatment allocation (open-label design). However, all efficacy assessments were conducted by independent dermatologists who did not participate in patient care and who remained blinded to treatment assignments throughout the study.

### Interventions

Treatment duration ranged from 12 to 16 weeks, with a follow-up period extending to 24 weeks post-treatment initiation. All patients concurrently received topical glucocorticoid ointment, calcipotriol ointment, or calcipotriol scalp solution as adjunctive therapies. The initial dosage of acitretin was 0.4 mg/kg/day, while cyclosporine was administered at 3 mg/kg/day (divided into two doses daily). Dose escalation was permitted to 0.5 mg/kg/day for acitretin and up to 5 mg/kg/day for cyclosporine if the PASI score reduction was less than 25% after 4 weeks of treatment. Medication doses could be reduced or discontinued at any point if adverse events (AEs) occurred, in accordance with established guidelines (12, 13). Patients received only topical therapies or phototherapy for 4 to 6 weeks during the screening period; no additional active systemic therapies were permitted during treatment, except emollients and topical treatments. Additional active treatments were allowed during the follow-up period, if clinically necessary.

**Figure 1:**
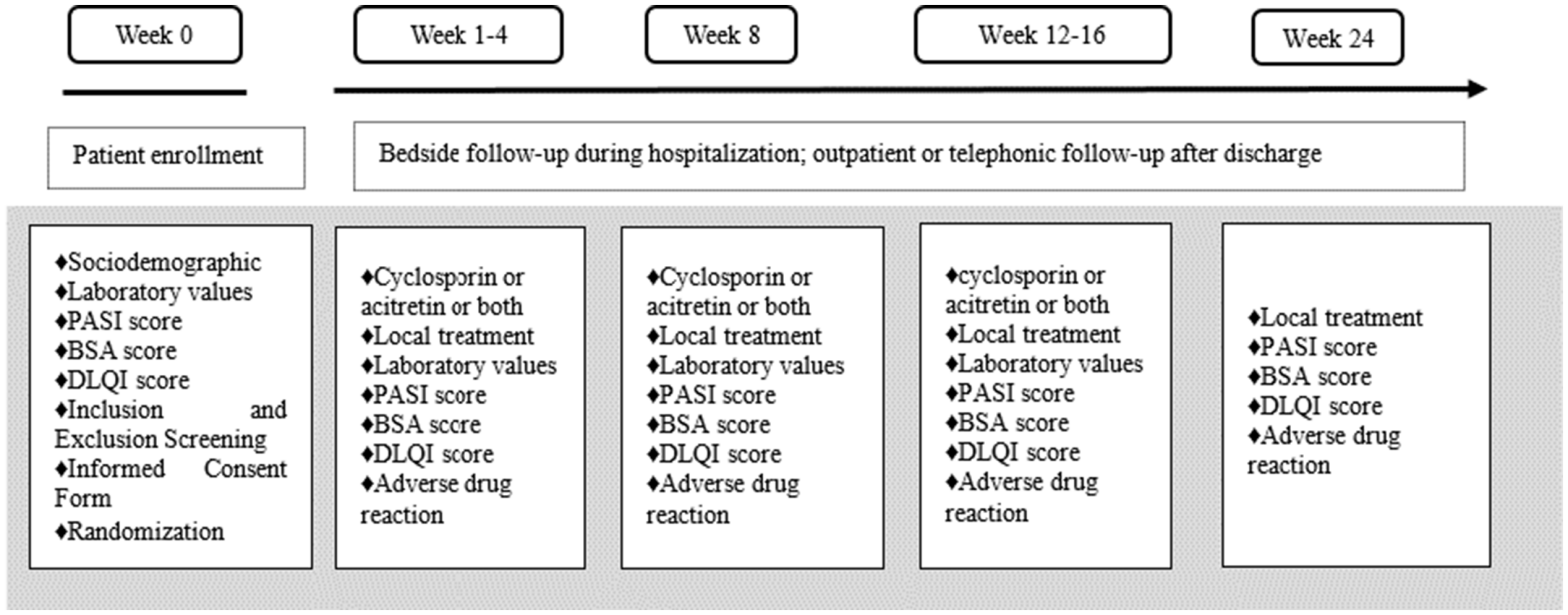
Depiction of the study timeline (24-week follow-up program).

To evaluate cyclosporine exposure, venous blood samples were collected immediately before the morning dose on Day 4 (i.e., prior to the 7th total dose, under twice-daily administration), representing the trough concentration (C0) at steady state. This timing was based on the pharmacokinetic profile of cyclosporine: with a half-life of approximately 6.3 h in healthy subjects (Sandimmun^®^, Novartis drug label), steady-state levels are expected to be reached within 4–5 half-lives (14, 15). Therefore, by Day 4, steady-state is considered to have been achieved for most patients. Cyclosporine concentrations were measured in both the combination therapy and cyclosporine monotherapy groups. Quantification was performed using liquid chromatography–tandem mass spectrometry (LC-MS/MS) with an AB SCIEX Triple Quad^™^ 4500MD system. The process of patient enrollment, treatment and follow-up is shown in Figure 1.

### Primary and secondary outcomes

The primary outcomes were the proportions of patients achieving at least a 75% (PASI75) and 90% (PASI90) reduction from baseline in the PASI. The key secondary outcome was the mean change in PASI score from baseline at each follow-up visit. Additional secondary outcomes included BSA, DLQI scores, and the incidence of AEs. All outcomes were assessed by independent dermatologists who were blinded to treatment allocation.

### Statistical analysis

Differences in mean PASI, BSA, and DLQI scores among the three treatment groups from baseline to week 24 were assessed using one-way analysis of variance (ANOVA) or non-parametric tests, as appropriate. Proportions of patients achieving PASI75 and PASI90 responses were compared between groups using the chi-square test or Fisher’s exact test, as appropriate. All statistical analyses were performed with SPSS software, version 20.0 (IBM Corp., Armonk, NY, United States). A two-sided *p*-value of <0.05 was considered statistically significant.

## Results

### Patient enrollment and baseline characteristics

A total of 351 patients were screened for eligibility, of whom 6 were excluded (2 owing to revised diagnoses not meeting inclusion criteria, 3 who declined to provide informed consent, and 1 who refused study medication). Consequently, 345 patients were randomized in a 1:1:1 ratio into the combination therapy group (*n* = 115), the acitretin monotherapy group (*n* = 115), and the cyclosporine monotherapy group (*n* = 115).

**Figure 2:**
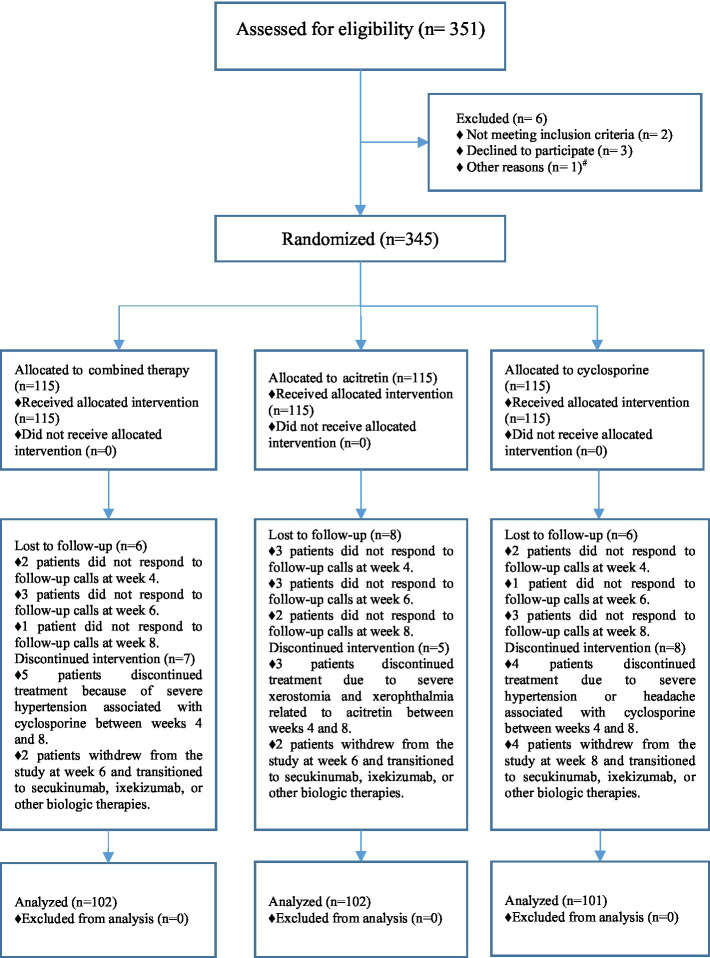
Patient enrollment and randomization flowchart. # One patient was withdrawn from the study prior to randomization and transitioned to methotrexate therapy due to a change in clinical condition.

During follow-up, 6 patients in the combination therapy group, 8 in the acitretin group, and 6 in the cyclosporine group were lost to follow-up. In addition, 7 patients in the combination therapy group, 5 in the acitretin group, and 8 in the cyclosporine group discontinued treatment (primarily because of adverse events or by switching to biologic therapy). Details of patient disposition are presented in Figure 2. Ultimately, 305 patients completed the study and were included in the final analysis: 102 in the combination therapy group, 102 in the acitretin group, and 101 in the cyclosporine group. Baseline characteristics of the patients are summarized in Table 1, showing no statistically significant differences in disease duration or baseline PASI scores among the groups.

**Table 1:** Baseline demographic and clinical characteristics of study participants.

Variables	Combined-therapy group (*n* = 102)	Acitretin group (*n* = 102)	Cyclosporine group (*n* = 101)	Total (*n* = 305)	*p*-value
Age (years)	45.42 ± 16.97	48.86 ± 15.87	43.23 ± 18.73	45.85 ± 17.32	0.065
Male sex	77 (75.5%)	73 (71.6%)	66 (65.3%)	216 (70.8%)	0.277
Weight (kg)	68.38 ± 12.65	70.21 ± 11.96	69.20 ± 17.46	69.26 ± 14.20	0.657
Body-mass index (kg/m^2^)	24.27 ± 3.88	24.80 ± 3.61	24.31 ± 4.89	24.46 ± 4.15	0.599
Duration of psoriasis (years)	11.76 ± 9.83	13.80 ± 10.33	13.55 ± 10.98	13.04 ± 10.40	0.312
PASI score	31.83 ± 10.15	29.43 ± 12.02	30.68 ± 9.06	30.65 ± 10.50	0.266
BSA score	0.63 ± 0.24	0.55 ± 0.19	0.57 ± 0.20	0.58 ± 0.21	0.018
DLQI score	21.21 ± 4.05	20.48 ± 4.68	21.17 ± 3.86	20.95 ± 4.21	0.385
The average dose of acitretin (mg/kg/day)	0.287 ± 0.061	0.411 ± 0.092	—	0.349 ± 0.100	<0.001
The average dose of Cyclosporine (mg/kg/day)	2.815 ± 0.462	—	3.387 ± 0.584	3.100 ± 0.598	<0.001
Plasma concentration of cyclosporine (ng/ml)	104.23 ± 37.26	—	161.67 ± 63.68	131.32 ± 58.80	<0.001

Data represent mean ± SD or the number of patients (%) for each group.

PASI, Psoriasis Area and Severity Index; BSA, Body Surface Area; DLQI, Dermatology Life Quality Index.

### Efficacy outcomes

**Figure 3:**
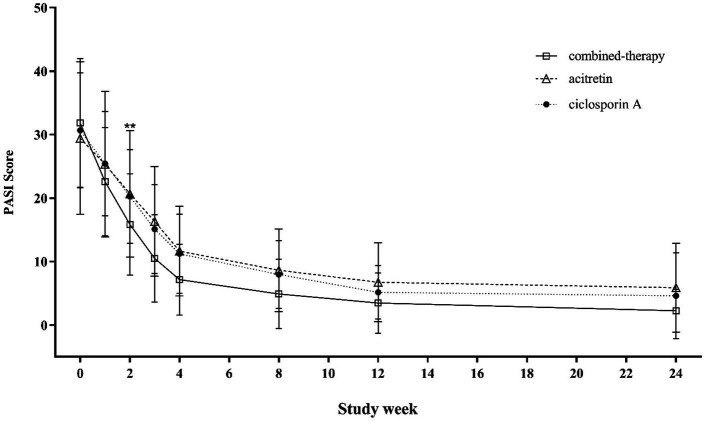
Trends in PASI scores from baseline to week 24 across treatment groups.

Figure 3 illustrates the changes in PASI scores from baseline (week 0) through week 24 across the three treatment groups. Between weeks 2 and 8, patients in the combination therapy group exhibited significantly lower mean PASI scores compared with those receiving either cyclosporine A or acitretin monotherapy (*p* < 0.001). From week 12 onward, overall differences in mean PASI scores among the three groups were attenuated and no longer statistically significant.

**Figure 4:**
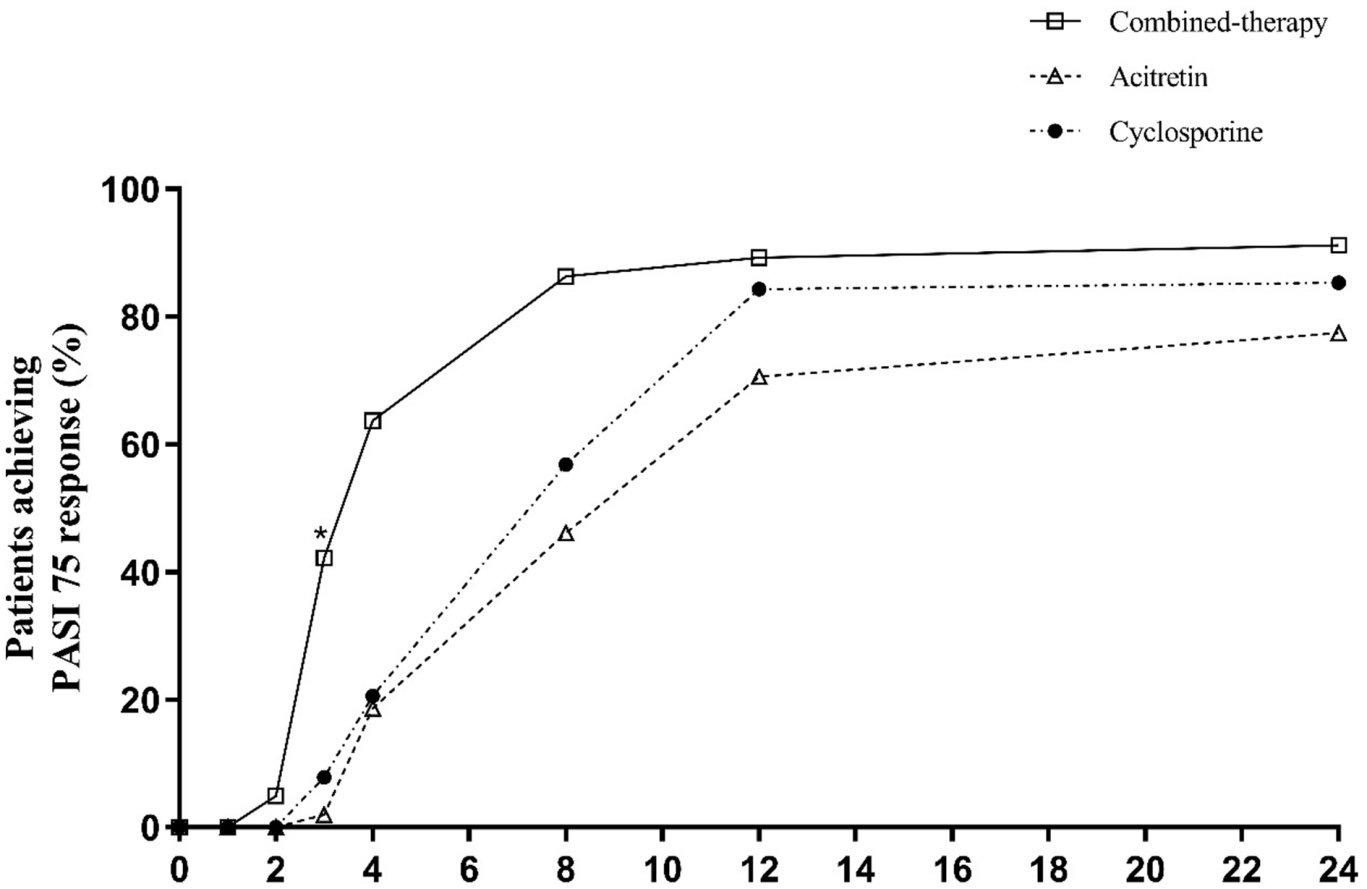
Percentage of patients achieving PASI75 response over time.

Regarding categorical outcomes, PASI75 responses showed early and sustained differences (Table 2 and Figure 4). By week 4, over 60% of patients in the combination therapy group had achieved PASI75, compared with fewer than 25% in either monotherapy arm (*p* < 0.001). The advantage of combination therapy was maintained through week 8 and week 12, when nearly 90% of patients in the combination group achieved PASI75, significantly higher than the acitretin and cyclosporine groups (*p* < 0.001). At week 24, PASI75 response rates remained highest in the combination therapy group (91.18%) compared with acitretin (77.45%) and cyclosporine (85.29%) (*p* = 0.022).

**Table 2:** PASI75 and pasi90 response rates by treatment group and study visit.

Variables	Combined-therapy group (*n* = 102)	Acitretin group (*n* = 102)	Cyclosporine group (*n* = 101)	*p*-value
Patients achieved PASI75 at week 1	0 (0.00%)	0 (0.00%)	0 (0.00%)	1.000
Patients achieved PASI75 at week 2	5 (4.90%)	0 (0.00%)	0 (0.00%)	0.012
Patients achieved PASI75 at week 3	43 (42.16%)	2 (1.96%)	8 (7.84%)	<0.001
Patients achieved PASI75 at week 4	65 (63.73%)	19 (18.63%)	21 (20.59%)	<0.001
Patients achieved PASI75 at week 8	88 (86.27%)	47 (46.08%)	58 (56.86%)	<0.001
Patients achieved PASI75 at week 12	91 (89.22%)	72 (70.59%)	86 (84.31%)	<0.001
Patients achieved PASI75 at week 24	93 (91.18%)	79 (77.45%)	87 (85.29%)	0.022
Patients achieved PASI90 at week 1	0 (0.00%)	0 (0.00%)	0 (0.00%)	1.000
Patients achieved PASI90 at week 2	0 (0.00%)	0 (0.00%)	0 (0.00%)	1.000
Patients achieved PASI90 at week 3	2 (1.96%)	0 (0.00%)	0 (0.00%)	0.331
Patients achieved PASI90 at week 4	22 (21.57%)	0 (0.00%)	3 (2.94%)	<0.001
Patients achieved PASI90 at week 8	47 (46.08%)	5 (4.90%)	10 (9.80%)	<0.001
Patients achieved PASI90 at week 12	68 (66.67%)	16 (15.69%)	31 (30.39%)	<0.001
Patients achieved PASI90 at week 24	79 (77.45%)	35 (34.31%)	60 (58.82%)	<0.001

Data represent the number of patients (%) for each group.

PASI, Psoriasis Area and Severity Index.

**Figure 5:**
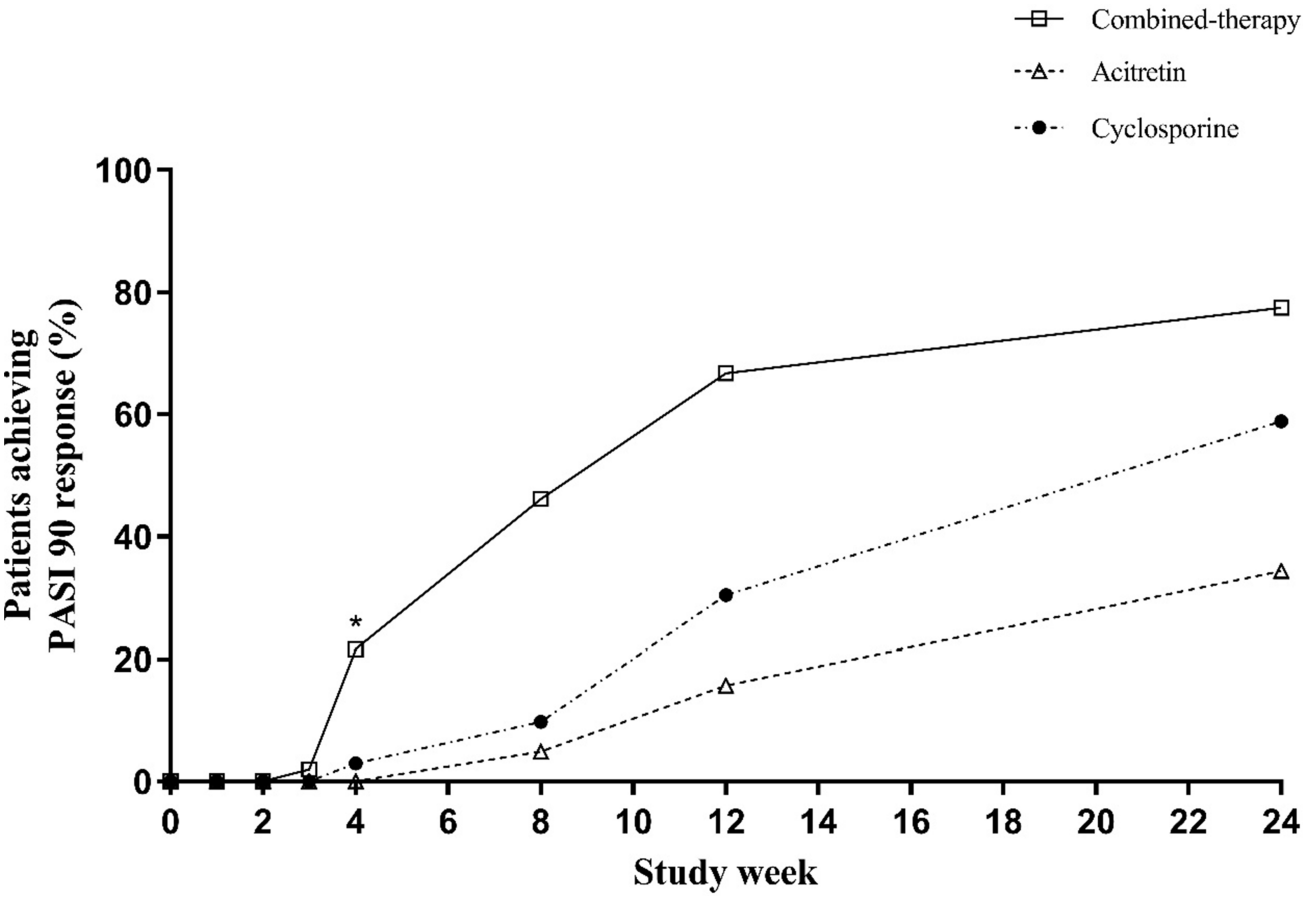
Percentage of patients achieving PASI90 response over time.

For PASI90 (Table 2 and Figure 5), early separation was also observed. By week 4, one-fifth of patients in the combination therapy group had already achieved PASI90, whereas responses were rare in the monotherapy groups. These early advantages were maintained at week 12, two-thirds of patients in the combination group reached PASI90 compared with ~15–30% in the monotherapy groups (*p* < 0.001). At week 24, PASI90 responses remained highest in the combination therapy group (77.45%), compared with 34.31 and 58.82% in the acitretin and cyclosporine groups, respectively (*p* < 0.001).

**Figure 6:**
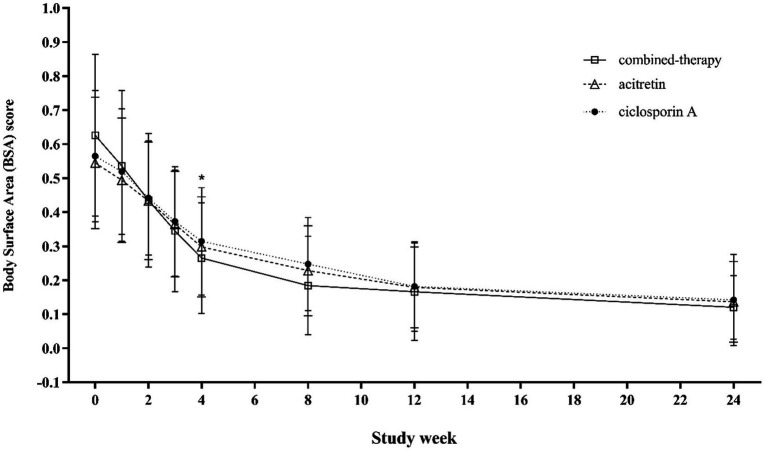
Trends in BSA scores from baseline to week 24 across treatment groups.

At baseline, mean BSA involvement was 0.55 ± 0.19 in the acitretin group, 0.63 ± 0.24 in the combination group, and 0.57 ± 0.20 in the cyclosporine group (*p* = 0.018) (Table 1), indicating that patients in the combination group had slightly greater disease burden at study entry. From week 4 onward, BSA improved significantly in all three groups, with the combination group showing greater reductions compared with both monotherapy groups (week 4: 0.265 ± 0.162 vs. 0.298 ± 0.147 and 0.314 ± 0.158, respectively; *p* < 0.001). These between-group differences in BSA were maintained through week 8, consistent with the pattern observed for PASI responses. By weeks 12–16, as clinical symptoms improved and many patients tapered or discontinued the study drug, BSA scores converged and no longer showed significant differences among groups (Figure 6).

**Figure 7:**
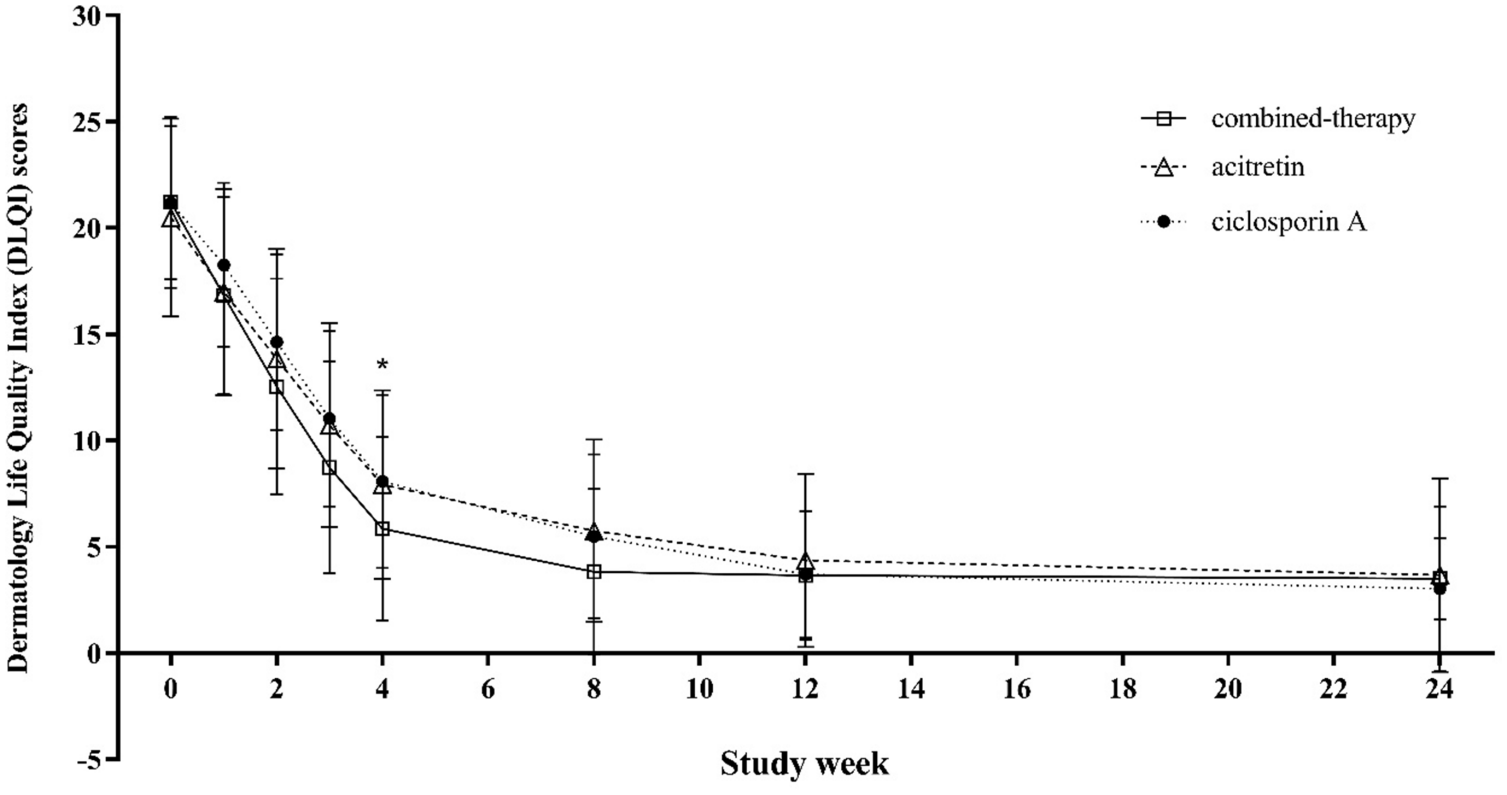
Trends in DLQI scores from baseline to week 24 across treatment groups.

Similarly, DLQI scores decreased markedly in all groups during treatment, reflecting improved quality of life. The greatest improvement was observed in the combination therapy group during the first 8 weeks, but differences among groups were not statistically significant after weeks 12–16 of treatment and remained comparable during follow-up to week 24 (*p* = 0.260) (Figure 7).

Dose escalation after 4 weeks was required for 13 patients in the acitretin group, 10 in the cyclosporine group, and 5 in the combination group. After 24 weeks of follow-up, relapse occurred in 15 patients in the acitretin group, 12 in the cyclosporine group, and 6 in the combination group.

Plasma cyclosporine concentrations were significantly lower in the combination therapy group (104.23 ± 37.26 ng/mL) than in the cyclosporine monotherapy group (161.67 ± 63.68 ng/mL, *p* < 0.001). The mean acitretin dose was also lower in the combination group (0.287 ± 0.061 mg/kg/day) compared with the acitretin group (0.411 ± 0.092 mg/kg/day, *p* < 0.001), as was the mean cyclosporine dose (2.815 ± 0.462 vs. 3.387 ± 0.584 mg/kg/day, *p* < 0.001) (Table 1).

### Safety and adverse events

At least one AE was reported by 49 patients in the acitretin group (48.04%), 34 patients in the cyclosporine group (33.66%), and 37 patients in the combination therapy group (36.27%) (Table 3). Most AEs were mild to moderate and resolved within 1–2 weeks after treatment discontinuation.

**Table 3:** Summary of adverse events during the 24-week study period.

Variables	Combined-therapy group (*n* = 102)	Acitretin group (*n* = 102)	Cyclosporine group (*n* = 101)	*p*-value
Patients with AE	37 (36.27%)	49 (48.04%)	34 (33.66%)	0.064
Hypertension	10 (9.80%)	2 (1.96%)	13 (12.87%)	0.015
Hepatic abnormalities	15 (14.71%)	12 (11.77%)	9 (8.91%)	0.427
Dyslipidemia	7 (6.86%)	18 (17.66%)	7 (6.93%)	0.013
Dry lips/dry eyes/dry skin	1 (0.98%)	12 (11.77%)	1 (0.99%)	<0.001
Headache	1 (0.98%)	0 (0.00%)	0 (0.00%)	0.999
Lumbar pain	0 (0.00%)	0 (0.00%)	1 (0.99%)	0.999
Renal dysfunction	0 (0.00%)	0 (0.00%)	1 (0.99%)	0.999
Diarrhea	1 (0.98%)	0 (0.00%)	1 (0.99%)	0.999
Alopecia	0 (0.00%)	1 (0.98%)	0 (0.00%)	0.999
Nausea/vomiting	1 (0.98%)	0 (0.00%)	0 (0.00%)	0.999
Discontinued study due to AE^a^	1 (0.98%)	3 (2.94%)	1 (0.99%)	0.459
Patients with ≥1 serious AE^b^	0 (0.00%)	1 (0.98%)	0 (0.00%)	0.999

Data represent the number of patients (%) for each group.

AE, adverse events.

a Severe hypertension in the combined-therapy and cyclosporine groups; Grade 3 alanine aminotransferase elevation (peak 203 U L^−1^) in three patients in the acitretin group. All laboratory abnormalities resolved within 1–4 weeks after treatment interruption.

b One patient in the acitretin arm developed severe alopecia after 3 months of therapy.

Hypertension was significantly more common in the cyclosporine group (13 of 101, 12.87%) than in the acitretin (2 of 102, 1.96%) and combination groups (10 of 102, 9.80%; *p* = 0.015). Three patients required antihypertensive treatment, with a mean cyclosporine dose of 3.10 ± 0.60 mg/kg/day in both cyclosporine-containing groups. Blood pressure returned to normal after cyclosporine discontinuation, and antihypertensive medications were concurrently withdrawn.

Dry lips, dry eyes, or dry skin were significantly more frequent in the acitretin group (12 of 102, 11.77%) compared with the cyclosporine (1 of 101, 0.99%) and combination groups (1 of 102, 0.98%; *p* < 0.001). Dyslipidemia occurred in 18 patients in the acitretin group (17.65%), compared with 7 in the cyclosporine group (6.93%) and 7 in the combination group (6.86%; *p* = 0.013). Hepatic abnormalities were observed in 15 patients (14.71%) in the combination group, 12 patients (11.77%) in the acitretin group, and 9 patients (8.91%) in the cyclosporine group (*p* = 0.427). These events were transient, rarely required additional medication, and resolved after drug discontinuation.

## Discussion

Patients with psoriasis often experience significant psychological distress and difficulties in social interactions (16). Moreover, psoriasis imposes a substantial economic burden globally, with direct and indirect costs ranging from 74 to 98 billion dollars annually in the United States and Asia (17, 18). Major challenges for elderly or low-income psoriasis patients include increased medical comorbidities, polypharmacy, and limited access to biologics due to high costs. Furthermore, biologics are contraindicated in patients with HIV, hepatitis B virus infection, or tuberculosis (19). Therefore, investigating the clinical efficacy and safety of traditional therapies, such as phototherapy, topical treatments, acitretin, cyclosporine, and methotrexate, remains critical.

For moderate-to-severe psoriasis, monotherapy with either phototherapy or systemic traditional medications often fails to achieve or maintain sufficient remission. This study demonstrated that both acitretin and cyclosporine monotherapies were effective, while the combination therapy significantly improved treatment outcomes for patients with moderate-to-severe psoriasis. Acitretin is considered one of the safest traditional FDA-approved systemic agents for psoriasis, especially with prolonged use exceeding 1 year (20). Unlike methotrexate or cyclosporine, acitretin does not significantly increase infection risks due to immunosuppression. Nevertheless, acitretin generally exhibits lower efficacy compared to methotrexate, cyclosporine, or biologics (21). Cyclosporine rapidly improves severe psoriasis flares (22); however, adverse events such as elevated blood pressure and headaches remain common complaints (22).

Currently, the combined use of acitretin and cyclosporine for moderate-to-severe psoriasis remains controversial. Numerous case studies support combined therapy using low-dose acitretin and cyclosporine, achieving prolonged remission (23, 24). A meta-analysis reported clearance rates of 39 to 100% for combination therapies compared to 2 to 86% for monotherapies (25). However, cases of failed combination therapy in erythrodermic psoriasis patients have also been documented (26). Caution is warranted when combining retinoids with cyclosporine, as both drugs are metabolized via the cytochrome P450 system, potentially increasing cyclosporine plasma concentrations. Additionally, both drugs may elevate plasma triglyceride levels, as observed in our study; hence, lipid monitoring is recommended.

Our study found that the combined use of cyclosporine and acitretin rapidly reduced PASI scores within 3 to 8 weeks. Moreover, combination therapy allowed lower dosages of both drugs (acitretin from 0.411 ± 0.092 mg/kg/day to 0.287 ± 0.061 mg/kg/day, *p* < 0.001; cyclosporine from 3.387 ± 0.584 mg/kg/day to 2.815 ± 0.462 mg/kg/day, *p* < 0.001), achieving superior efficacy at lower cyclosporine plasma concentrations, thus reducing adverse events. These findings offer important implications for providing cost-effective therapeutic options for psoriasis, particularly benefiting economically disadvantaged patients who require long-term treatment (27).

We observed that adverse events such as cheilitis, xerosis, and elevated plasma lipids occurred in approximately half of patients treated with acitretin, more frequently than in patients treated with cyclosporine alone or combined therapy. Consistent with previous studies, liver enzyme elevations associated with acitretin were reversible upon dose reduction or discontinuation (28–30). Notably, tetracycline antibiotics and hepatotoxic medications should be used cautiously with acitretin due to risks of pseudotumor cerebri (31) and increased hepatotoxicity (32). Cyclosporine effectively controls severe psoriasis rapidly but should be used cautiously in elderly patients or those with renal impairment due to nephrotoxicity risks (33).

Importantly, although the incidence of hepatic events was higher in the combination group than in the monotherapy group, the difference was not statistically significant (*p* = 0.427). No dose-dependent relationship with cyclosporine was observed; rather, these events may have been related to acitretin exposure. The mean acitretin dose among patients with hepatic abnormalities in the combination group was 0.35 mg/kg/day, compared with 0.42 mg/kg/day in the acitretin monotherapy group. Previous studies have suggested that cyclosporine may inhibit CYP3A4-mediated metabolism of acitretin (7, 34), potentially increasing susceptibility to hepatic injury, although this requires further investigation. Moreover, all patients who experienced hepatotoxic adverse events returned to normal within approximately 1–2 weeks after a reduction in the dosage of the investigational drug.

Overall, both acitretin and cyclosporine were administered at significantly lower mean doses in the combination group than in their respective monotherapies. Except for hepatic abnormalities, the incidence of adverse events in the combination group was lower than with acitretin and generally comparable to cyclosporine, supporting an acceptable overall safety profile for the combination regimen.

### Limitations

This study has several limitations. First, the relatively small sample size and the exclusion of patients with erythrodermic psoriasis, pustular psoriasis, and psoriatic arthritis limit the generalizability of the findings. The absence of radiographic assessments and evaluation of nail involvement further restricts the scope of disease characterization. In addition, the dose and potency of topical glucocorticoid ointments were not evaluated, which may have influenced treatment outcomes.

Second, the open-label design, while necessary for practical reasons, introduced potential performance bias and detection bias in patient-reported outcomes. To mitigate this, the primary efficacy endpoints were assessed by blinded dermatologists, reducing the risk of detection bias.

Finally, baseline BSA differed across treatment groups, with patients in the combination therapy arm presenting with greater disease involvement at study entry (*p* = 0.018). Although randomization was otherwise balanced and the differences were relatively small, this imbalance may have introduced bias when comparing treatment effects. To address this, efficacy was primarily assessed using relative measures of improvement (e.g., PASI75 and PASI90 response rates), which are less affected by baseline severity than absolute scores. Nonetheless, the possibility that baseline BSA differences influenced the magnitude of between-group comparisons cannot be excluded, and our conclusions should therefore be interpreted with this limitation.

## Conclusion

Cyclosporine A combined with acitretin produced faster and more pronounced clinical improvement than either drug alone in moderate-to-severe plaque psoriasis, with responses emerging by weeks 3–4 and peaking at week 12. The combination permitted lower cyclosporine doses and plasma concentrations, while also lessening cheilitis, xerosis, hypertension, and dyslipidaemia seen with monotherapy. As an all-oral, relatively affordable option, it may benefit patients who cannot access biologics. Larger, well-controlled studies are still needed to confirm these results, define an optimal cyclosporine concentration window, establish the minimum effective acitretin dose, and clarify the regimen’s long-term safety.

## Data Availability

The raw data supporting the conclusions of this article will be made available by the authors, without undue reservation.
